# What Shall We Eat, and What Shall We Drink?

**Published:** 1855-04

**Authors:** 

**Affiliations:** Cincinnati


For Hall's Journal of Health.

### "WHAT SHALL WE EAT, AND WHAT SHALL WE DRINK?"

BY PROFESSOR CLEAVELAND, OF CINCINNATI.

> "The mind shall banquet though the body pine,
> Fat paunches make lean pates; and dainty bits
> Make rich their ribs, but banker out their wits."
>
> LOVE'S LABOR LOST

THE fable of the vulture which preyed thirty thousand years upon the liver of Prometheus, because he stole heavenly fire, seems replete with truth in more senses than one, for those who try to obtain the fires of youth or health in an unnatural degree, but search *not Heaven*—and steal the unnatural fires of alcoholic drinks with which to quicken their passions, have their livers eaten upon as with vultures, and like their great prototype, *their* livers also are not consumed by the gnawings, but continue undiminished and even enlarged by the action of the disease.

Many men perform the wonderful necromantic trick of "digging their grave with their own teeth," and others still more strangely seem to glide down their own throats into air—and *thus*, perhaps it *may* be, a part of them have the "*Throat ail.*"

The Friend, or Quaker, it is true, oftentimes has a fair rotundity of person, but usually along with it he carries a breadth of shoulder and a placid face which indicate a clear conscience and a stomach not punished with dyspepsia. And of this people it is estimated that more than half their number live to the age of forty-five, and that at least one in ten attain the age of four score years. Temperance and virtue are among the greatest panaceas yet discovered.

As an evidence of the truthfulness of the play-writer, Sir Isaac Newton found it necessary, while composing his work on optics, to confine himself to a diet of vegetables, and to drink nothing but water. John Locke was told that his intellectual powers *increased* with his increasing years, but he said it was because he became more and more plain and simple in his diet, and that his work on the *Human Understanding* was the result—not so much of a clear head as of a clean stomach. President Edwards, the theologian, made a remark very similar in his Diary, in which is recorded that the smallest amount of plain food compatible with bodily health was the most conducive to sprightliness and activity of mind. The younger Addison, not only in his beautiful Lines to Temperance, but elsewhere and often, bore similar testimony.

Dr. Franklin and Dr. Rush, agree with Dr. Cheyne in his statement, "that he who would have a clear head, must have a clean stomach." Cameades of Greece acted on this principle, for before he would dispute with Chrysippus the Stoic, he took a dose of physic to cleanse out the alimentary canal. He would have done better not to allow its obstruction.

Dr. Hosack says, "like the mark upon the forehead of Cain after the commission of his crime, so do the "bubuckles, whelks and *rosy drops*," denote the suicidal practice of the habitual dram drinker, and indicate the malignant spirit that reigns within.

Garth says,

> "The first physicians by debauch were made,
> Excess began, and still sustains the trade."

It will be perceived that the most of these authorities lived before the Maine Law was dreamed of by Neal Dow, and hence they spoke as well of intemperance in eating, as of intemperance in drinking. Cadogan, in his work on Gout, speaks as well of stimulating condiments as of stimulating drinks; and it may be that these and other improper articles of food being more fully indulged in by males than females, in the West Indies, and the Southern States, is one reason why so many widows in those sections are so anxiously awaiting the advent of their second or third husband.

But the use of tobacco, and crude or improperly cooked, or high seasoned food is seldom indulged in to a great extent without the accompanying inebriant.

Each demand the other, and each demands the physician, lean pates, and the sexton. Let us all then seriously consider, what we shall eat and what we shall drink.

